# Effects of Ethylene Propylene Diene Monomer (EPDM)-Based Polar Macromolecular Compatibilizers on the Low-Temperature Properties of Fluoroelastomer/EPDM Rubber Blends

**DOI:** 10.3390/molecules29235522

**Published:** 2024-11-22

**Authors:** Gen Liu, Faxin Du, Zhangjun Yao, Guangzhao Li, Wen Kuang, Chongyu Zhu, Yi Liu, Honglin Chen, Fumei Wang, Ce Zhou, Xueli Wei, Wenyan Wang, Rui Han

**Affiliations:** 1 Key Laboratory of Materials and Surface Technology (Ministry of Education), School of Materials Science and Engineering, Xihua University, Chengdu 610039, China; liugen@mail.xhu.edu.cn (G.L.); faxindu_study@163.com (F.D.); yaozj1997@163.com (Z.Y.); kuangw1027@163.com (W.K.); honglin_chen23@163.com (H.C.); fumei_wang0919@163.com (F.W.); wwyandmmy@163.com (W.W.); ruihan_harry@163.com (R.H.); 2 Engineering Research Center of Intelligent Air-Ground Integration Vehicle and Control (Xihua University), Ministry of Education, Chengdu 610039, China; 3 College of Chemistry and Chemical Engineering, Donghua University, Shanghai 201620, China; czhu@dhu.edu.cn; 4 Meishan CRRC Brake Science & Technology Co., Ltd., Meishan 620010, China; 13890385880@163.com (Y.L.); ssx3514@163.com (C.Z.); 13548245154@163.com (X.W.)

**Keywords:** compatibilizers, fluorine rubber, rubber blends, low-temperature resistance

## Abstract

Integrating rubber with superior low-temperature capabilities, such as ethylene propylene diene monomer (EPDM), is a strategic approach to bolster the low-temperature performance of fluoroelastomer (FKM). However, FKM and EPDM are thermodynamically incompatible. This work synthetized three EPDM-based polar macromolecular compatibilizers, epoxidized EPDM (EPDM-EP), 2,2-trifluoroethylamine-grafted epoxidized EPDM (EPDM-TF), and 2,4-difluorobenzylamine-grafted epoxidized EPDM (EPDM-DF), to enhance the compatibility between FKM and EPDM. These compatibilizers were subsequently incorporated into FKM/EPDM rubber blends. The results revealed that the glass transition temperature (T_g_) of FKM/EPDM decreased by 1.3 °C, 2.68 °C, and 2.78 °C, respectively, upon the addition of 10 phr of EPDM-EP, EPDM-TF, or EPDM-DF. Moreover, the T_g_ of the two phases converged. The tensile strength, elongation at break, and tear strength of the FKM/EPDM rubber blends were also enhanced by the inclusion of these compatibilizers. Notably, EPDM-TF and EPDM-DF exhibited remarkable compatibilization effects due to an increase in polarity. This research not only sheds light on the potential for developing new compatibilizers but also paves the way for innovative applications of FKM and its derivatives.

## 1. Introduction

Fluoroelastomer (FKM) boasts high chemical stability, weather resistance, oxidation resistance, oil resistance, and minimal permeability [1]. Consequently, FKM is extensively utilized in sectors such as aerospace, automotive, petrochemical, and electronics for applications including solvent-resistant washing rollers, heat exchanger seals, pump components, O-rings, diaphragm seals, and various other components [2,3,4]. Despite these advantages, FKM’s performance is compromised at low temperatures, which restricts its use in cold environments. At extremely low temperatures, the diminished thermal motion of rubber molecules leads to a significant reduction in molecular chain elasticity due to the increased rotational barrier in the carbon chain caused by fluorine atoms [5].

Researchers have undertaken numerous studies to enhance the low-temperature resistance of FKM. For instance, Moni et al. [6] developed FKM composites with improved low-temperature resistance by incorporating reduced graphene oxide (mRGO) into FKM. An increase in mRGO content from 0.5 phr to 2 phr resulted in a 2.97 °C decrease in the glass transition temperature (T_g_) of FKM. Ma et al. augmented the low-temperature resistance of FKM by blending it with modified silicon-based nanoparticles, which led to a T_g_ reduction from −4.08 °C to −7.36 °C in the modified FKM. Furthermore, blending FKM with other low-temperature-resistant rubbers is an effective strategy to enhance its low-temperature performance. Commonly used blending rubbers include silicone rubber, ethylene propylene diene monomer (EPDM), and polyurethane rubber. These materials perform excellently at low temperatures and can significantly lower the T_g_ of FKM while enhancing its flexibility. Kim et al. [7] modified silicone rubber by blending it with FKM, observing a T_g_ decrease from −12.2 °C to −14.7 °C as the silica rubber content increased, although this came at the cost of a 45.5% reduction in tensile strength and a 52.5% decrease in elongation at break. Wu et al. created composite rubber by blending reinforced silicone rubber with FKM, which improved the low-temperature resistance of FKM, with the T_g_ dropping from −20 °C to −41 °C and the tensile strength decreasing by 31.25% at a test temperature of −20 °C. These findings underscore the importance of maintaining the mechanical stability of FKM while enhancing its low-temperature resistance.

EPDM, a copolymer of ethylene, propylene, and a third monomer (commonly a diene), exhibits exceptional low-temperature performance and aging resistance, making it ideal for applications in automotive sealing strips, building waterproofing materials, and electrical insulation [8,9]. When blended with FKM, EPDM not only enhances the low-temperature performance and processing characteristics of FKM/EPDM rubber blends but also reduces material costs while preserving the heat resistance and chemical resistance of the blends. However, the blending of FKM and EPDM often encounters issues such as poor compatibility due to differences in polarity, unsaturation, and crosslinking active sites [10,11].

This study focuses on the development of three types of EPDM-based polar macromolecular compatibilizers, EPDM-EP, EPDM-TF, and EPDM-DF, to enhance the polarity of EPDM. These compatibilizers were then added to FKM/EPDM rubber blends to create FKM/EPDM/EPDM-EP, FKM/EPDM/EPDM-TF, and FKM/EPDM/EPDM-DF blends. The study’s objective was to investigate the impact of these compatibilizers on the low-temperature resistance and mechanical properties of FKM/EPDM blends. It is anticipated that this research will provide innovative insights into the production of high-performance FKM rubber blends.

## 2. Results and Discussion

### 2.1. Composition and Structure of EPDM-EP, EPDM-TF and EPDM-DF

**Figure 1:**
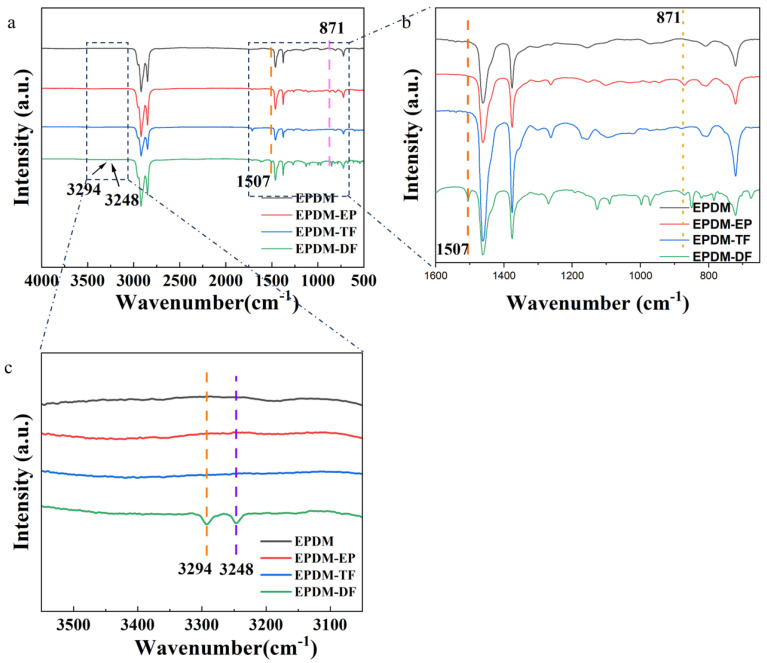
Comparison of infrared spectra of EPDM and spectra after modification; (**a**) infrared spectra comparison images of EPDM, EPDM-EP, EPDM-TF, and EPDM-DF; (**b**) enlarged image at 750–1600 cm^−1^; (**c**) enlarged image at 3050–3500 cm^−1^.

**Figure 2:**
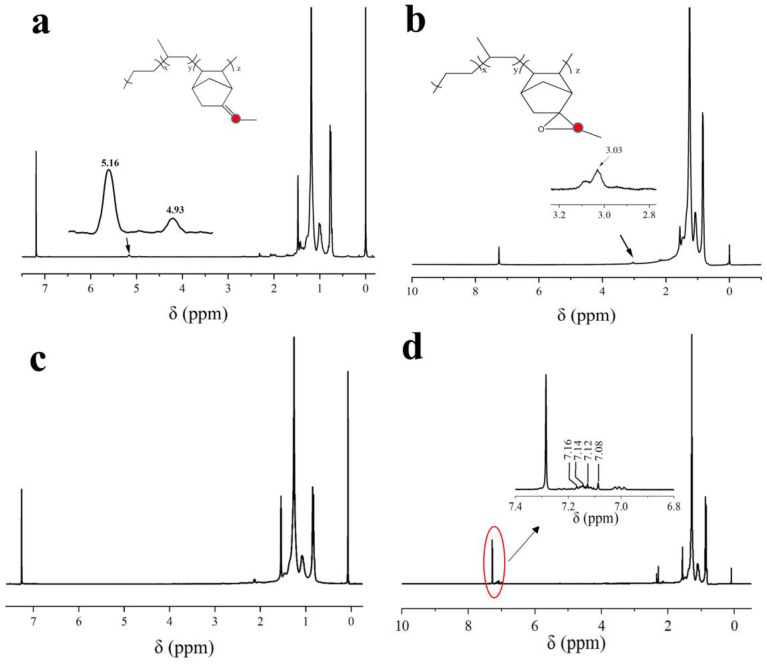
H-NMR images of EPDM and its modified compounds (**a**) EPDM; (**b**) EPDM-EP; (**c**) EPDM-TF; (**d**) EPDM-DF. Red dots in (**a**,**b**) indicate the hydrogen atoms corresponding to the peaks in the partially enlarged images. The red circle shows the position of the enlarged area within the wider spectrum.

Figure 1 presents a comparative analysis of the infrared spectra of EPDM, EPDM-EP, EPDM-TF, and EPDM-DF. In Figure 1a,b, a peak at 810 cm^−1^ corresponds to the C=C double bond on ENB (effect of load on the stick–slip phenomenon and wear behavior of EPDM). Following the chemical interaction between EPDM and t-BuOOH, a new peak emerges at 871 cm^−1^ (Figure 1b), indicative of the antisymmetric stretching vibration of the epoxy C-O-C [12]. This observation confirms the successful epoxidation of the C=C double bond on ENB within EPDM. The peaks at 3294 cm^−1^, 3248 cm^−1^, and 1505 cm^−1^ in Figure 1b,c correspond to the stretching and deformation vibrations of the secondary amino group on DF, signifying the successful grafting of DF onto EPDM-EP [13]. However, the characteristic absorption peak of the imine group is absent in the infrared spectra, likely due to the relatively low reaction degree between TF and EPDM, resulting in a weak characteristic absorption peak of the secondary amino group that is not visible in the infrared spectra. Subsequent H-NMR analysis will further substantiate the successful reaction between EPDM-EP and TF (Figure 2).

Figure 2 displays the H-NMR spectra of EPDM, EPDM-EP, and two EPDM fluorinated modifiers, EPDM-TF and EPDM-DF. In Figure 2a, the two resonance peaks at chemical shifts δ of 5.16 ppm and 4.93 ppm represent the hydrogen peaks of the E and Z configurations of the external double bond of bonding ENB [14]. The H-NMR curve of EPDM-EP exhibits a marked decrease in the resonance peak at 5.16 ppm and 4.93 ppm, coupled with the emergence of a new peak at 3.03 ppm, which corresponds to the vibrational peak of the hydrogen atom on the epoxy group [15], indicating that EPDM has been successfully epoxidized. In Figure 2c, it can be observed that the hydrogen resonance peaks at 5.16 ppm and 4.93 ppm on the double bond of EPDM, as well as the hydrogen resonance peak at 3.03 ppm on the epoxy group of EPDM-EP, have all vanished. In conjunction with the infrared spectra in Figure 1a, this confirms that TF has successfully reacted with EPDM. The new peak appearing at 7.08–7.16 ppm in the nuclear magnetic resonance hydrogen spectra of EPDM-DF (Figure 2d) corresponds to the hydrogen atom resonance peak of the benzene ring on the DF, and the absorption vibration peak of the hydrogen atom on the epoxy group also disappears at 3.03 ppm. Collectively, these results confirm that both TF and DF have successfully reacted with EPDM.

### 2.2. Effect of EPDM-TF and EPDM-DF on the Interface Structure of Blending Rubbers

**Figure 3:**
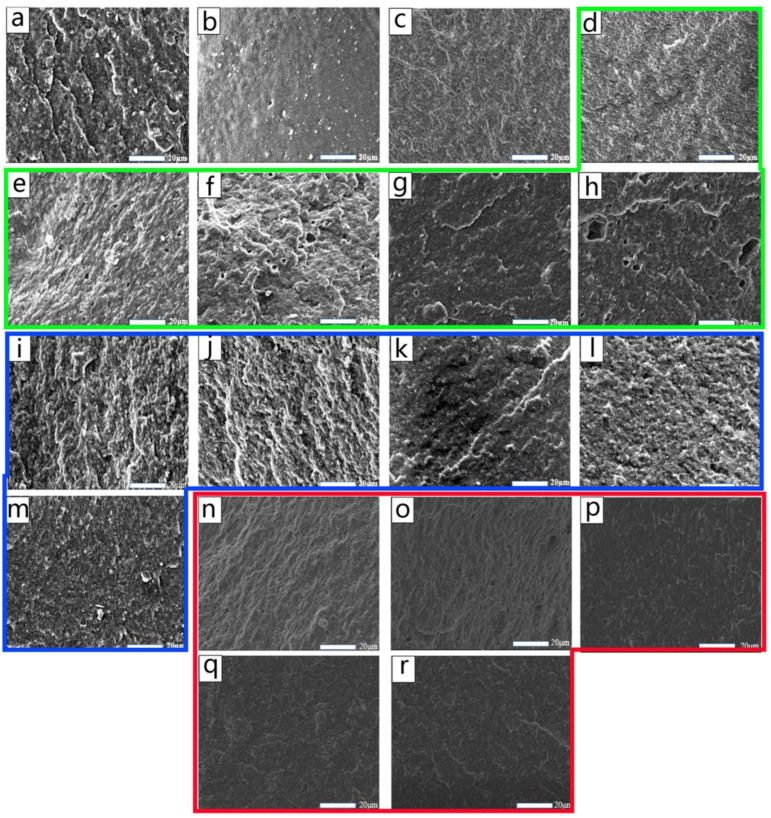
Cross-section SEM micrographs of FKM (**a**); EPDM (**b**); F/E (**c**); F/E/EPDM-EPy, y = 2, 4, 6, 8, 10 phr (**d**–**h**); F/E/EPDM-TFy, y = 2, 4, 6, 8, 10 phr (**i**–**m**); and F/E/EPDM-DFy, y = 2, 4, 6, 8, 10 phr (**n**–**r**).

Figure 3a–c display SEM images of FKM, EPDM, and the FKM/EPDM rubber blend, respectively. The surfaces of the FKM and EPDM rubbers appear uniform and flat, whereas the FKM/EPDM blend exhibits a heterogeneous dispersion and a rougher surface at the microscopic level. Upon incorporating the compatibilizer EPDM-EP, the surface of the blend rubber progressively smoothens (Figure 3d–h), with a marked reduction in the interfacial boundaries. The dispersed phase domains diminish in size, leading to a more uniform phase distribution. This suggests that EPDM-EP acts as an adhesive between FKM and EPDM, effectively lowering the interfacial tension [16,17] and enhancing the surface smoothness of the blend rubber. Figure 3i–m depict cross-sectional SEM micrographs of EPDM-TF with varying additions, while Figure 3n–r display cross-sectional SEM micrographs of EPDM-DF with varying additions. It is evident that a small quantity of EPDM-TF results in poor compatibility between the EPDM and FKM phases, hindering thorough mixing and yielding a rough interface morphology. However, when the EPDM-TF content reaches 6 phr or higher, the blend rubber’s interface becomes smoother, and the compatibility between the phases improves, leading to a more uniform FKM and EPDM blend with stronger interphase bonding. Comparing the results in Figure 3i–m with those in Figure 3c,n–r, the inclusion of EPDM-DF renders the blend rubber’s surface smoother, reduces roughness, and induces significant alterations in the phase structure. As the EPDM-DF dosage is increased further, the interphase becomes increasingly smooth, and the dispersed phase domains decrease and distribute evenly, signifying that EPDM-DF significantly enhances the compatibility within the FKM/EPDM blend system. The SEM findings indicate that EPDM-DF outperforms EPDM-TF in terms of interface compatibilization for FKM/EPDM rubber. This observation will be corroborated in subsequent mechanical property assessments.

### 2.3. The Impact of Compatibilizers on the Low-Temperature Properties and Compatibility of Blending Rubbers

**Figure 4:**
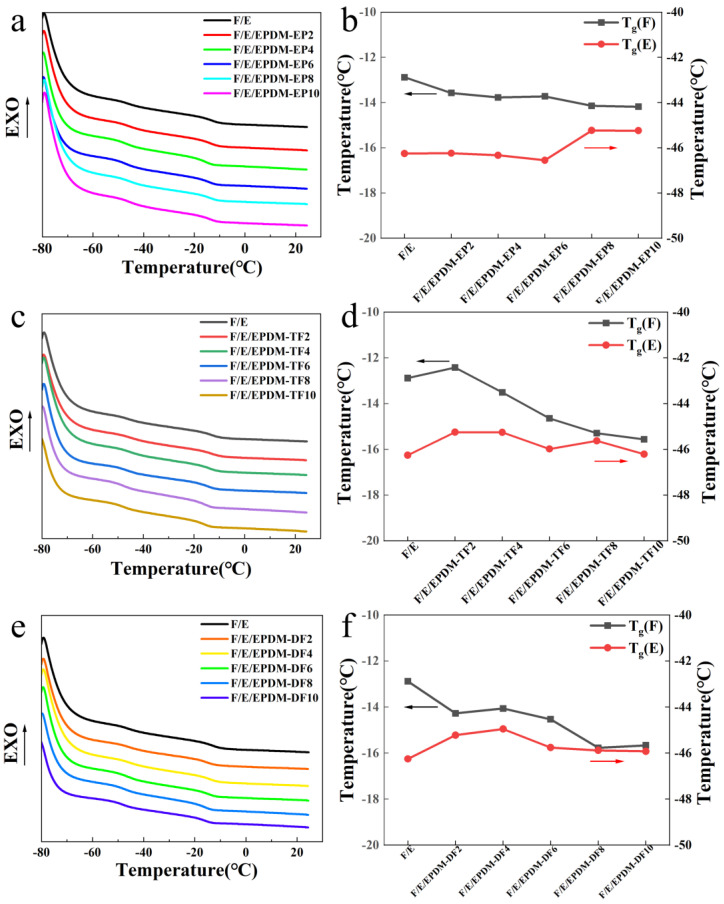
DSC curves of FKM/EPDM rubber blends incorporating EPDM-EPy (**a**); EPDM-TFy (**c**); and EPDM-DFy (**e**); respectively, y = 2, 4, 6, 8, 10 phr. Additionally, the variation in T_g_ of rubber blends with respect to EPDM-EP (**b**), EPDM-TF (**d**), or EPDM-DF (**f**) content. The arrow points to its corresponding vertical axis.

Figure 4 illustrates the DSC test curves and T_g_ change curves of FKM/EPDM blends following the addition of three types of compatibilizers at varying concentrations. As depicted in Figure 4a,c,e, the FKM/EPDM blends exhibit two glass transition zones, which arise from the inadequate compatibility between FKM and EPDM. With an increased amount of the three compatibilizers, there is a downward trend in the T_g_ of the FKM phase within the blends. Specifically, the addition of 10 phr of EPDM-EP, 10 phr of EPDM-TF, and 10 phr of EPDM-DF to the FKM/EPDM blends results in a decrease in the T_g_ of the FKM phase by 1.3 °C, 2.68 °C, and 2.78 °C, respectively. This can be attributed to the incorporation of polar groups into EPDM, which hinders the crystallization of FKM. As shown in Figure 4b,d,f, the T_g_ values of FKM and EPDM move closer together with the addition of EPDM-EP, EPDM-TF, or EPDM-DF, indicating that EPDM-TF/DF possesses compatibilizing effects on the blend rubber. It is hypothesized that the varying polarity levels among the chemical groups introduced by the macromolecular compatibilizers contribute to different degrees of Tg reduction. Moreover, TF and DF share more structural similarities with FKM. The synthetically derived compatibilizers EPDM-TF and EPDM-DF play a crucial role in improving compatibility by forming a linkage between the hydrophobic EPDM and the fluorinated FKM. This facilitates enhanced adhesion and interaction at the interface. Consequently, the incorporation of EPDM-TF and EPDM-DF leads to a greater reduction in Tg compared to EPDM-EP. Since all three compatibilizers undergo epoxy ring-opening reactions during either preparation or vulcanization, the further introduction of fluorinated polar groups into EPDM by reacting with TF and DF beforehand significantly improves the compatibility between EPDM and FKM.

**Figure 5:**
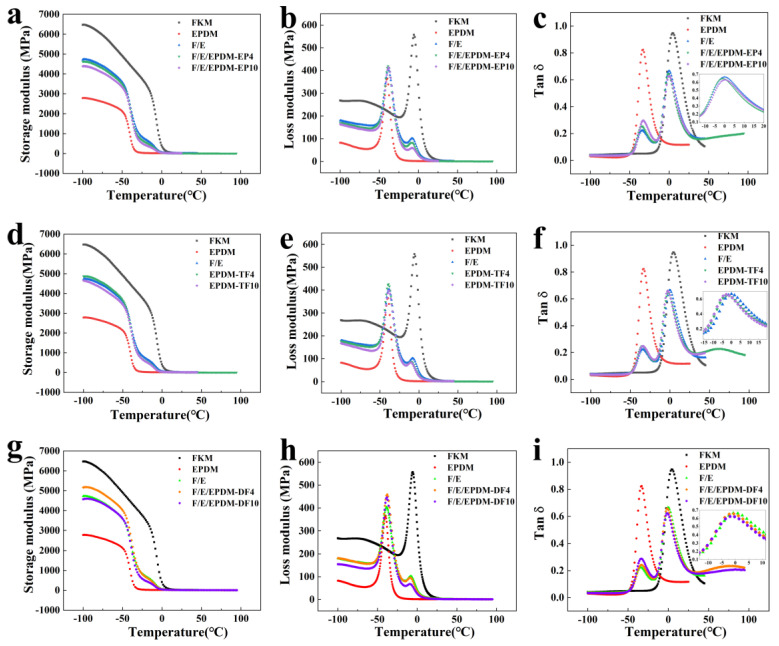
Storage modulus (**a**), loss modulus (**b**), and tan δ (**c**) of FKM, EPDM, F/E, F/E/EPDM-EP4, and F/E/EPDM-EP10; storage modulus (**d**), loss modulus (**e**), and tan δ (**f**) of FKM, EPDM, F/E, F/E/EPDM-TF4, and F/E/EPDM-TF10; storage modulus (**g**), loss modulus (**h**), and tan δ (**i**) of FKM, EPDM, F/E, F/E/EPDM-DF4, and F/E/EPDM-DF10.

The data presented in Figure 5 and Table 1 reveal that an increase in the concentration of EPDM-EP leads to a reduction in the Young’s modulus of the blended rubber. This phenomenon can be attributed to the lower stiffness of EPDM compared to FKM, as well as to the fact that EPDM-EP possesses properties akin to those of EPDM. Consequently, an elevated EPDM-EP content is tantamount to an increased EPDM content. Furthermore, as depicted in Figure 5c, an increase in EPDM-EP results in a shift of the EPDM T_g_ peak towards higher temperatures, while the FKM T_g_ peak simultaneously shifts towards lower temperatures. According to Table 1, the T_g_ difference between FKM/EPDM/EPDM-EP10 is 33.31 °C, which represents a decrease of 1.13 °C from the 34.44 °C difference observed between FKM/EPDM. This observation indicates that an increased EPDM-EP content brings the T_g_ values of the blended rubber closer together, thereby enhancing the compatibility between FKM and EPDM.

In Figure 5d–f, the Young’s modulus, loss modulus, and loss factor test curves of blending rubber with varying amounts of EPDM-TF added in FKM/EPDM are depicted. The addition of compatibilizer EPDM-TF results in a decreasing trend in the Young’s modulus of the blending rubber, attributed to the lower stiffness of EPDM compared to FKM. An increase in the EPDM-TF dosage can be interpreted as an increase in the EPDM dosage, thus leading to a decrease in the Young’s modulus of the blending rubber. Following the addition of 10 phr of EPDM-TF, the T_g_ (F) was observed to be −2.8 °C, indicating a reduction of 2.01 °C when compared to that of the pure sample FKM/EPDM. Consequently, the T_g_ difference between the two phases decreased from 34.44 °C to 31.54 °C. A similar trend in change is also evident in the DSC test results (Figure 4d).

Figure 5g–i illustrates the test curves for the Young’s modulus, loss modulus, and loss factor of the blending rubber following the incorporation of EPDM-DF into FKM/EPDM. The trends observed in the changes in the Young’s modulus and loss modulus are consistent with those observed upon the addition of EPDM-TF, which may be attributed to the similar properties of the two compatibilizers. However, it is important to note that the addition of EPDM-DF results in a shift of the T_g_ (F) of the blending rubber towards lower temperatures, while T_g_ (E) also exhibits a tendency to move in the same direction. After the addition of 4 phr of EPDM-DF, the T_g_ difference between the two phases decreased from 34.44 °C to 32.01 °C; following the addition of 10 phr of EPDM-DF, the T_g_ (F) was found to be −1.14 °C, representing a decrease of 1.07 °C when compared to that of the pure sample FKM/EPDM. The DSC test results also displayed a corresponding trend in change (Figure 5f). Collectively, the glass transition peaks of the FKM/EPDM blend rubber exhibit a convergence trend following the addition of the three compatibilizers. These findings suggest that both EPDM-TF and EPDM-DF are more effective in enhancing the compatibility between FKM and EPDM than that noted for EPDM-EP, while also improving the low-temperature resistance of FKM, postulating that the higher polarity of EPDM-TF and EPDM-DF, compared to EPDM-EP, contributes to their superior low-temperature performance.

### 2.4. The Effect of EPDM-EP, EPDM-TF, and EPDM-DF on the Mechanical Properties of Blending Rubbers

**Figure 6:**
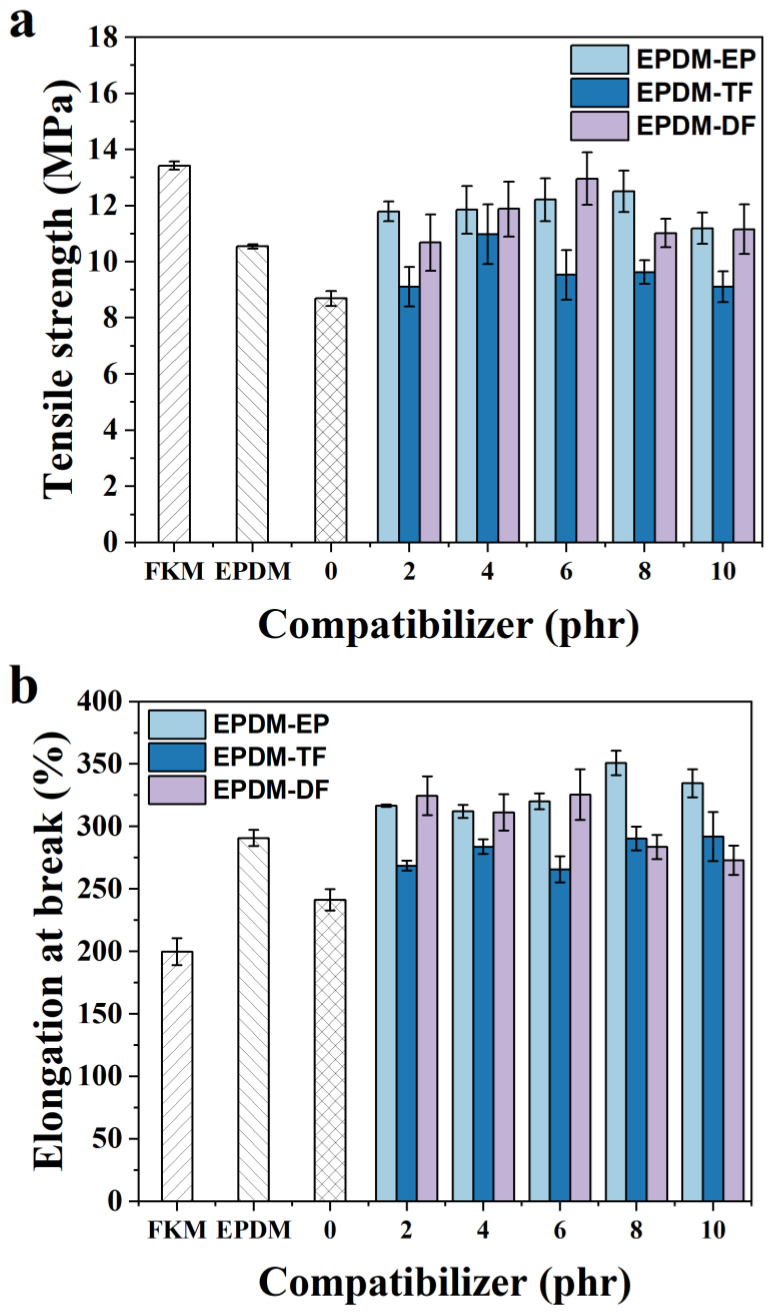
The influence of varying concentrations of EPDM-EP, EPDM-TF, and EPDM-DF on the tensile strength (**a**) and elongation at break (**b**) of rubber materials.

Figure 6a,b illustrates the comparative effects of incorporating three different compatibilizers on the tensile strength and elongation at break of the FKM/EPDM blends. As observed in Figure 6a, the addition of compatibilizers in various proportions has led to an enhancement in the tensile strength of the rubber. Specifically, the inclusion of 8 phr of EPDM-EP resulted in a tensile strength of 12.51 MPa for the FKM/EPDM rubber, which represents a 43.96% increase relative to the blend without EPDM-EP. Furthermore, the addition of 4 phr of EPDM-TF to the FKM/EPDM blend led to the maximum tensile strength, exhibiting a 26.35% improvement over the blend without any compatibilizer. Additionally, with the incorporation of 6 phr of EPDM-DF into the FKM/EPDM blend, the tensile strength reached its peak and increased by 49.14%. This can be explained by the fact that the compatibilizers facilitate better intermolecular interactions and a more uniform phase distribution, thereby enhancing the overall performance of the blend. When the amount of compatibilizer reaches a certain level, it effectively covers all interfaces that require improved compatibility. However, as the amount of compatibilizer continues to increase, there may be a point at which the compatibilizers can no longer efficiently cover the increased interfacial area or manage the interactions between the growing FKM or EPDM domains. Beyond a certain threshold, the additional compatibilizer may not provide significant improvements in compatibility and can lead to a leveling off or even a decrease in mechanical properties. This can occur due to over-modification or the introduction of unnecessary complexity in the blend, which can result in phase separation or the formation of larger microdomains that act as stress concentrators [18,19].

In Figure 6b, it is evident that an increase in the amount of compatibilizer leads to a gradual increase in the elongation at break for the F/E/EPDM-EP and F/E/EPDM-TF blends. Conversely, the F/E/EPDM-DF blend initially experiences an increase, followed by a decrease in elongation at break. In terms of tensile properties, EPDM-DF demonstrates a superior compatibilization effect on the FKM/EPDM system when compared to that of EPDM-EP or EPDM-TF. This observation aligns with the outcomes obtained from infrared spectroscopy and H-NMR imaging, which indicate a higher degree of reaction between DF and EPDM-EP than between TF and EPDM-EP. Moreover, the fluorine-modified epoxidized EPDM possesses a greater polarity. Consequently, the EPDM-DF compatibilizer, prepared through the modification with DF, exhibits enhanced compatibilization properties.

**Figure 7:**
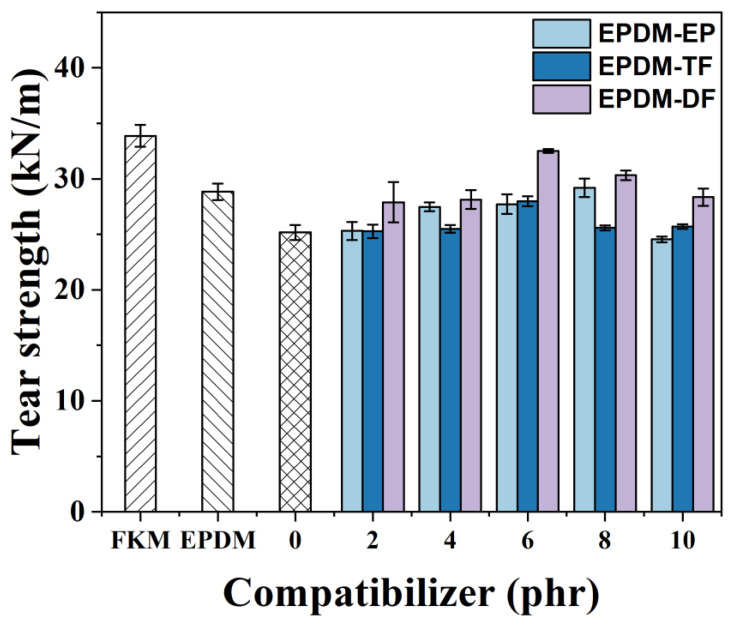
The effect of variation of EPDM-EP, EPDM-TF, and EPDM-DF on the tear strength of FKM/EPDM rubber blends.

Figure 7 illustrates a comparative assessment of the tear strength exhibited by blending rubbers that have been treated with varying quantities of three different compatibilizers. As the concentration of these compatibilizers is increased, there is an observable trend where the tear strength initially rises and subsequently declines. Specifically, the addition of a compatibilizer at a concentration of 6 phr results in the F/E/EPDM-DF blend achieving its maximum tear strength, which is measured at 32.51 kN/m. This represents a 29.16% enhancement in tear strength when compared to that of the FKM/EPDM blend that has not been treated with any compatibilizer. The SEM analysis suggests that this improvement in tear properties is attributed to the EPDM-DF compatibilizer’s superior ability to enhance the compatibility between the FKM and EPDM phases within the blending system. Furthermore, the compatibilizer also plays a role in reducing the tensile forces that exist between these two phases.

**Figure 8:**
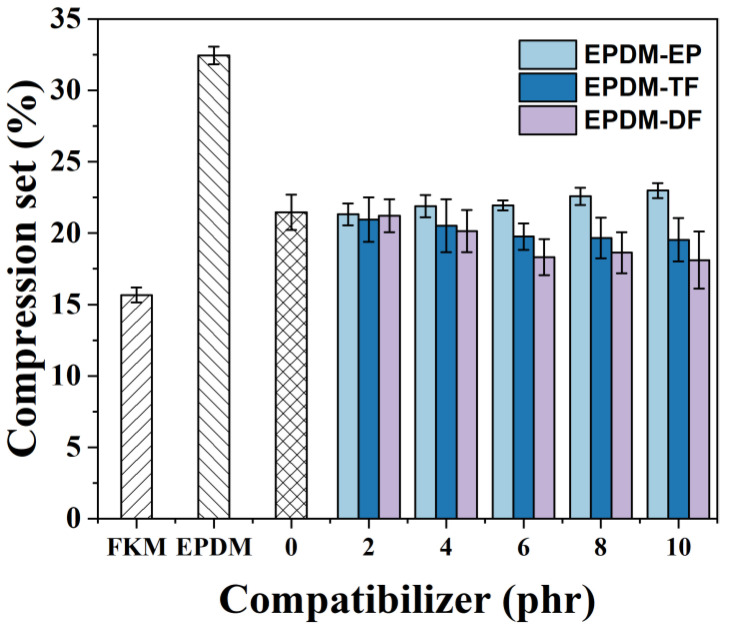
The effect of variation of EPDM-EP, EPDM-TF, and EPDM-DF on the compression set of FKM/EPDM rubber blends.

As depicted in Figure 8, it is evident that an increase in the content of either EPDM-TF or EPDM-DF leads to a decrease in the EN compression set rate of the FKM/EPDM blends. When 6 phr or more of EPDM-DF/TF is incorporated into the FKM/EPDM rubber, the EN compression set rate of the resulting blends ceases to decrease and remains relatively stable. The addition of 10 phr of either EPDM-TF or EPDM-DF results in a reduction of the blend rubber’s compression set by 1.92% and 3.35%, respectively, when compared to that of the FKM/EPDM blend without any compatibilizer. This improvement is attributed to the EPDM-DF/TF compatibilizers’ ability to enhance interfacial properties, increase stiffness, and ultimately improve the blend rubber’s resistance to external forces, as confirmed by SEM test results.

**Figure 9:**
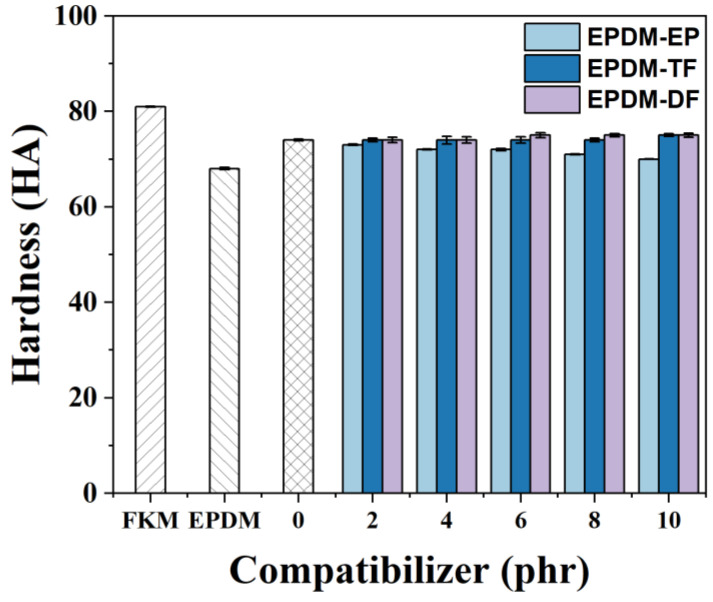
The effect of variation of EPDM-EP, EPDM-TF, and EPDM-DF on the hardness of FKM/EPDM rubber blends.

Figure 9 presents a comparative analysis of the hardness values of FKM, EPDM, and their respective blends following the incorporation of varying quantities of compatibilizers. An elevation in the EPDM-EP content is associated with a marginal reduction in the hardness of the blended rubber. Notably, the hardness of the blend only exhibited a 1 HA increase when 10 phr of EPDM-TF was introduced. In contrast, the addition of 6 phr of EPDM-DF led to a comparable 1 HA enhancement in hardness. The graphical data suggest that the inclusion of these three compatibilizers exerts a minimal influence on the overall hardness of the blended rubber.

## 3. Materials and Methods

### 3.1. Materials

FKM (CA-1300) was procured from Chenguang Kemu Fluorine Materials (Shanghai, China) Co., Ltd. EPDM (J-4045-E), featuring 5-Ethylidene-2-norbornene (ENB) as the third monomer, which was sourced from Jilin Petrochemical Co., Ltd. (Jilin, China). Trifluoroethylamine (TF) and difluorobenzylamine (DF), both of analytical grade, were obtained from Shanghai Aladdin Biochemical Technology Co., Ltd. (Shanghai, China). The carbon blacks utilized were Thermax^®^ N990 and Corax^®^ N330, provided by Cancarb (Medicine Hat, AB, Canada). Benzyltriphenylphosphorus chloride (BPP, analytical grade), hexafluorobisphenol A (BPAF, analytical grade), and sulfur (S, analytical grade) were purchased from Zigong Tianlong Chemical Co., Ltd. (Zigong, China). Additional materials included stearic acid (325 mesh) from Sanyi Chemical Co., Ltd. (Qingdao, China); palm wax (analytical grade) from KAHLWAX (Trittau, Germany); N-cyclohexyl-2-benzothiazolesulfonamide (CBS, analytical grade), tetramethylthiuram disulfide (TMTD, analytical grade), magnesium oxide (MgO, analytical grade), zinc oxide (ZnO, analytical grade), calcium hydroxide (Ca(OH)_2_, analytical grade), anhydrous magnesium sulfate (MgSO_4_, analytical grade), xylene (analytical grade), methanol (analytical grade), tert-butyl hydroperoxide (t-BuOOH, analytical grade), molybdenum trioxide (MoO_3_, analytical grade), zinc chloride (ZnCl_2_, analytical grade), trichloromethane (analytical grade), sodium chloride (NaCl, analytical grade), and sodium bicarbonate (NaHCO_3_, analytical grade) from Shanghai McLean Biochemical Technology Co., Ltd. (Shanghai, China).

### 3.2. Preparation of EPDM-EP by EPDM Epoxy Modification

**Figure 10:**
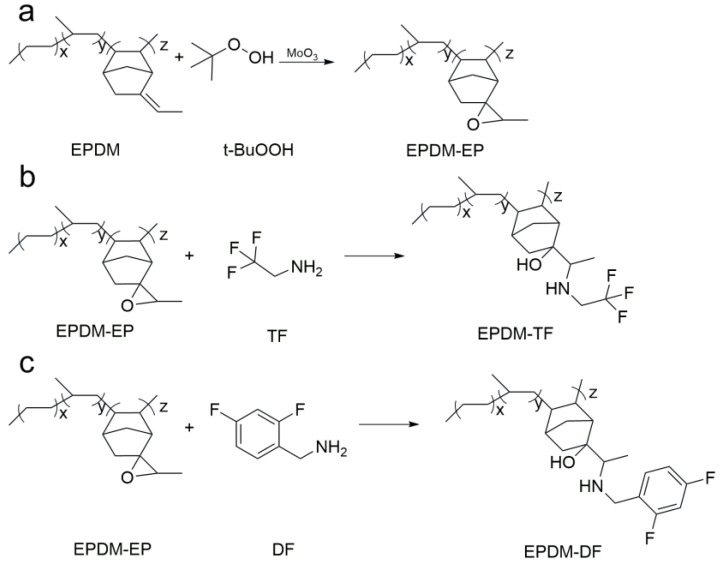
Schematic diagram of the EPDM epoxy modification reaction (**a**) and TF (**b**)/DF (**c**) grafted onto EPDM-EP.

A three-necked flask was charged with 100 mL of xylene and 3 g of EPDM. Once the EPDM was fully dissolved and the mixture cooled to room temperature, 0.15 mL of tert-butyl hydroperoxide (TBHP) and 0.15 g of molybdenum trioxide (MoO_3_) were introduced into the flask. A condensation reflux reaction was then carried out at 90 °C for 8 h. Following the completion of the reaction and subsequent cooling to room temperature, the reaction mixture was poured into an excess of methanol to precipitate the product, which was subsequently filtered. The yellow precipitate was washed with deionized water and dried to a constant weight in an oven set at 40 °C. The specific reaction process is illustrated in Figure 10a.

### 3.3. Preparation of EPDM-TF/DF by Grafting Modification of EPDM-EP

A three-necked flask was charged with 100 mL of xylene and 2.5 g of EPDM-EP, which was heated until the EPDM-EP had completely dissolved. Upon reaching room temperature, the solution was cooled, and then 0.5 mL of trifluoroethylamine (TF) and 0.06 g of anhydrous zinc chloride were added as catalysts. A condensation reflux reaction was conducted at 100 °C for 6 h. After the reaction was completed and the solution had cooled to room temperature, it was poured into an excess of methanol for precipitation and filtration. The precipitate was then repeatedly rinsed with an excess of deionized water. Finally, the obtained precipitate was dried in an oven at 40 °C until it reached a constant weight [20,21]. The reaction process is illustrated in Figure 10b.

The preparation process of EPDM-DF was analogous to that of EPDM-TF. Specifically, 0.4 mL of difluorobenzylamine (DF) and 0.06 g of anhydrous zinc chloride were added to the fully dissolved EPDM-EP/xylene solution. This mixture was then subjected to a condensation reflux reaction at 100 °C for 6 h. Following precipitation and filtration with an excess of methanol, the product was washed repeatedly with deionized water. Ultimately, the product was dried in an oven at 40 °C to achieve a constant weight [22]. The specific reaction process is illustrated in Figure 10c.

### 3.4. Preparation of FKM/EPDM Rubber Blends

The basic formulation of the FKM/EPDM rubber blends is detailed in Table 2. Initially, FKM and EPDM were blended for 3 min (60 rpm, 130 °C) using a TSE-30A mixer from Nanjing Reya Fost Polymer Equipment Co., Ltd. (Nanjing, China). Next, half of the additives were introduced, and the blending continued for an additional 8 min (120 rpm, 130 °C). The remaining additives were then added, and the mixing process was prolonged for another 5 min (60 rpm, 130 °C). The blended product was subsequently removed and cut into smaller pieces. These shredded pieces were reintroduced into the mixer for an additional 3 min (60 rpm, 50 °C), followed by the addition of the vulcanizing agent and vulcanization auxiliaries to complete the mixing process, which was continued for another 5 min. The final mixture was processed in an open mixer (S (X) K-160, Shanghai Tuolin Light Chemical Machinery Factory Co., Ltd. (Shanghai, China)), with a roll spacing initially set at 3 mm, undergoing 10 cycles. The roll spacing was then reduced to 2 mm, and another 10 cycles were performed. After mixing, the product was allowed to rest for 24 h. It was then placed in a hot press for vulcanization at 170 °C and 12 MPa for a duration of 7 min. Finally, the product was cured in an oven for 12 h. The FKM/EPDM blends without the addition of EPDM-EP, EPDM-DF, or EPDM-TF are referred to as F/E. For the F/E/EPDM-Xy blends, “X” denotes the type of EPDM-based polar macromolecular compatibilizer, while “y” indicates the additive amounts.

### 3.5. Characterization

Fourier transform infrared spectroscopy (FTIR, TENSOR 27, Bruker, Billerica, MA, USA) was employed to analyze the chemical groups and states of EPDM-EP, EPDM-TF, and EPDM-DF. The molecular structures of these compounds were determined using proton nuclear magnetic resonance spectrometry (H-NMR) (AVANCE NEO 400, Bruker, Billerica, MA, USA), with chloroform-d serving as the solvent. Differential scanning calorimetry (DSC, TA25, TA Instruments, New Castle, DE, USA) was utilized to measure the glass transition temperature (T_g_) of the rubbers. Approximately 10–15 mg of the blends was placed in an alumina crucible and cooled to −80 °C at a rate of 10 °C/min, held at this temperature for 1 min, and subsequently heated to 30 °C at a rate of 20 °C/min. Dynamic mechanical analysis (DMA, TAQ80, TA Instruments, USA) was conducted in a single cantilever mode to assess the dynamic mechanical properties of the rubbers. The sample dimensions were 10 mm × 9 mm × 2 mm, and the testing temperature ranged from −100 °C to 100 °C, with a heating rate of 3 °C/min. The frequency was set at 1 Hz. The surface and cross-sectional morphologies of the rubbers were examined using a scanning electron microscope (SEM) (Gemini 500, Zeiss, Oberkochen, Germany). The samples were immersed in liquid nitrogen to induce brittle fracture, and the cross-sections were sputter-coated with gold prior to imaging. Tensile tests were performed in accordance with the standard ISO 37:2017 [23] using a universal testing machine. The tensile speed was set at 500 mm/min, and dumbbell-shaped specimens (75 mm × 2 mm) with a gauge length of 20 mm were utilized. The tensile strength and percentage elongation at break were recorded. Tear resistance performance was assessed according to ISO 34-1:2022 [24] using right-angled uncut specimens (100 mm × 2 mm) at room temperature. Compression set characteristics were determined following ISO 815-1:2019 [25] using Method B. Cylindrical samples with a diameter of 13 ± 0.5 mm and a height of 6.3 ± 0.3 mm were employed. The appropriate height limiter was selected based on the sample hardness. The cylindrical sample and limiter were assembled in a fixture and placed in a high-temperature oven at 150 °C for 24 h. Post-cooling for 30 min, the heights of the samples were measured. Shore hardness tests were conducted in compliance with the standard ISO 7619-1:2004 [26] at room temperature. Tensile tests, tear resistance tests, compression set, and Shore hardness were each conducted five times for each sample type.

## 4. Conclusions

To address the issues of poor compatibility between FKM and EPDM in FKM/EPDM blending rubber, this study employs tert-butyl hydroperoxide as the initiator and molybdenum trioxide as the catalyst to perform epoxidation modification on EPDM, preparing EPDM-EP. Subsequently, TF and DF are used to graft-modify EPDM-EP, resulting in the synthesis of the new compatibilizers EPDM-EP, EPDM-TF, and EPDM-DF. FTIR and NMR results confirm the successful preparation of these compatibilizers. Their effects on the interface structure, low-temperature resistance, compatibility, and mechanical properties of FKM/EPDM blending rubber have been thoroughly investigated. After adding 10 phr of EPDM-EP, EPDM-TF, and EPDM-DF, the glass transition temperature (T_g_) of the blending rubber decreased by 1.3 °C, 2.68 °C, and 2.78 °C, respectively. Additionally, the two glass transition peaks of the blending rubber tended to merge, indicating improved compatibility between the FKM and EPDM phases. In terms of mechanical properties, the tensile strength of the EPDM-TF and EPDM-DF blending rubbers increased by 26.35% and 49.14%, respectively, compared to the results for the blends without compatibilizers. The elongation at break also improved by 21% and 34.80%, while the tear strength increased by 2.82 kN/m and 7.34 kN/m, respectively. As the content of the compatibilizers increased, the EN compression set of the blending rubber decreased, further demonstrating the positive impact of these compatibilizers on the blend’s performance. This study confirms that grafting fluorinated polar groups onto EPDM is an effective strategy to enhance the performance of FKM/EPDM blending rubbers, providing valuable insights for improving the low-temperature performance of FKM.

## Figures and Tables

**Table 1:** The influence of EPDM-EP, EPDM-TF, and EPDM-DF addition on the T_g_ of rubber blends.

Sample	T_g_ (F) (°C)	T_g_ (E) (°C)	T_g_ (F)-T_g_ (E)
FKM	4.74	/	/
EPDM	/	−33.51	/
F/E	−0.07	−34.51	34.44
F/E/EPDM-EP4	−0.76	−34.57	33.81
F/E/EPDM-EP10	−0.13	−33.44	33.31
F/E/EPDM-TF4	−2.08	−34.71	32.63
F/E/EPDM-TF10	−2.8	−34.34	31.54
F/E/EPDM-DF4	−1.44	−33.45	32.01
F/E/EPDM-DF10	−1.14	−33.31	32.17

Note: T_g_ was calculated based on the peak position of the loss factor (tan δ).

**Table 2:** Formula table of bisphenol AF/BPP/S/CBS/TMTD vulcanization system (phr).

Materials	Dosages (phr)	Materials	Dosages (phr)
FKM	70	Stearic acid	0.3
EPDM	30	Carnauba wax	1
Carbon black N990	21	BPAF	2.5
MgO	2.1	BPP	0.6
Ca(OH)_2_	4.2	S	0.3
Carbon black N330	9	CBS	0.3
ZnO	1.5	TMTD	0.15

## Data Availability

The datasets generated and/or analyzed during the current study are available from the corresponding author upon reasonable request.

## References

[1] Gao J.C., Wu Y.D., Liu Y.Y., Zhou Z.M., Fan Z.Q., Wei T.T., Yin D.W., Zhou F., Fu H., Jin H.L. (2024). Preparation of high performance fluoroelastomers by modifying carbon fiber with PEI and fluorinated coupling agent. Eur. Polym. J..

[2] Simon A., Pepin J., Berthier D., Méo S. (2023). Degradation mechanism of FKM during thermo-oxidative aging from mechanical and network structure correlations. Polym. Degrad. Stab..

[3] Tang S., Li J., Wang Z., Zhang L.Q. (2023). Design and Synthesis of Novel Bio-Based Polyester Elastomer with Tunable Oil Resistance. Macromol. Rapid Commun..

[4] Shi D.C., Cai L., Zhang C.Z., Chen D.F., Pan Z.H., Kang Z., Liu Y., Zhang J.J. (2023). Fabrication methods, structure design and durability analysis of advanced sealing materials in proton exchange membrane fuel cells. Chem. Eng. J..

[5] Wang S.H., Hou M.C., Ma K., Li Z.W., Geng H., Zhang W.W., Li N. (2022). Research on the Influence of Extremely Cold Environment on the Performance of Silicone Rubber and Fluorinated Silicone Rubber. Polymers.

[6] Kim D.H., Hwang S.H., Kim B.S. (2012). The effects of technological compatibility for silicone rubber/fluororubber blends. J. Appl. Polym. Sci..

[7] Wu W.L., Li X. (2019). Wear and thermal properties of carbon fiber reinforced silicone rubber/fluorine rubber composites. J. Rubber Res..

[8] Colom X., Carrillo-Navarrete F., Saeb M.R., Marin M., Formela K., Cañavate J. (2023). Evaluation and rationale of the performance of several elastomeric composites incorporating devulcanized EPDM. Polym. Test..

[9] Spanheimer V., Jaber G.G., Katrakova-Krüger D. (2023). Ground Tire Rubber Particles as Substitute for Calcium Carbonate in an EPDM Sealing Compound. Polymers.

[10] Tom M., Thomas S., Seantier B., Grohens Y., Mohamed P.K., Ramakrishnan S., Kuriakose J. (2023). Aspects of Dynamic Mechanical Analysis in Polymeric Materials.

[11] Toczek K., Lipinska M., Pietrasik J. (2021). Smart TPE Materials Based on Recycled Rubber Shred. Materials.

[12] Liu B., Wu W. (2018). Nonisothermal crystallization kinetics of poly(butylene terephthalate)/epoxidized ethylene propylene diene rubber/glass fiber composites. Polym. Eng. Sci..

[13] Dietz J.P., Lucas T., Gross J., Seitel S., Brauer J., Ferenc D., Gupton B.F., Opatz T. (2021). Six-Step Gram-Scale Synthesis of the Human Immunodeficiency Virus Integrase Inhibitor Dolutegravir Sodium. Org. Process Res. Dev..

[14] Ali A., Akram M.A., Guo Y., Wu H., Liu W., Khan A., Liu X., Fu Z., Fan Z. (2019). Ethylene–propylene copolymerization and their terpolymerization with dienes using ansa-Zirconocene catalysts activated by borate/alkylaluminum. J. Macromol. Sci. Part A.

[15] de Roo C.M., Kasper J.B., van Duin M., Mecozzi F., Browne W. (2021). Off-line analysis in the manganese catalysed epoxidation of ethylene-propylene-diene rubber (EPDM) with hydrogen peroxide. RSC Adv..

[16] Junior A.J.A., Saron C. (2023). Mechanical recycling of expanded polystyrene and tire rubber waste as compatibilized and toughened blends. J. Appl. Polym. Sci..

[17] Zhu Z., Chen H., Zhu X., Sang Z., Sukhishvili S.A., Uenuma S., Ito K., Kotaki M., Sue H.-J. (2023). Strengthening and toughening of polybenzoxazine by incorporation of polyrotaxane molecules. Compos. Sci. Technol..

[18] Khalf A.I., Nashar D.E.E., Maziad N.A. (2010). Effect of grafting cellulose acetate and methylmethacrylate as compatibilizer onto NBR/SBR blends. Mater. Des..

[19] Debbah I., Krache R., Aranburu N., Etxeberria A., Pérez E., Benavente R. (2020). Influence of abs type and compatibilizer on the thermal and mechanical properties of pc/abs blends. Int. Polym. Process..

[20] Malani R.S., Malshe V.C., Thorat B.N. (2022). Polyols and polyurethanes from renewable sources: Past, present and future—Part 1: Vegetable oils and lignocellulosic biomass. J. Coat. Technol. Res..

[21] Lu H., Dun C., Jariwala H., Wang R., Cui P.Y., Zhang H.P., Dai Q.G., Yang S., Zhang H.C. (2022). Improvement of bio-based polyurethane and its optimal application in controlled release fertilizer. J. Control. Release.

[22] Lou W.X., Dai Z.D., Jiang P.P., Zhang P.B., Bao Y.M., Gao X.W., Xia J.L., Haryono A. (2022). Development of soybean oil-based aqueous polyurethanes and the effect of hydroxyl value on its properties. Polym. Adv. Technol..

[23] (2017). Rubber, Vulcanized or Thermoplastic-Determination of Tensile Stress-Strain Properties.

[24] (2022). Rubber, Vulcanized or Thermoplastic-Determination of Tear Strength-Part 1: Trouser, Angle and Crescent Test Pieces.

[25] (2019). Rubber, Vulcanized or Thermoplastic-Determination of Compression Set-Part 1: At Ambient or Elevated Temperatures.

[26] (2004). Rubber, Vulcanized or Thermoplastic-Determination of Indentation Hardness-Part 1: Durometer Method (Shore Hardness).

